# High-MOI induces rapid CRISPR spacer acquisition in *Sulfolobus* from an *acr* deficient virus

**DOI:** 10.17912/micropub.biology.000664

**Published:** 2022-11-08

**Authors:** Yuvaraj Bhoobalan-Chitty, Xiaoxiao Duan, Xu Peng

**Affiliations:** 1 Department of Biology, University of Copenhagen, Copenhagen N, Denmark

## Abstract

Spacer acquisition, the first step in CRISPR-Cas adaptive immunity, plays a critical role in establishing and strengthening host defense against mobile genetic elements (MGEs). Here we present a host-virus system, where an increase in the multiplicity of infection (MOI), of a CRISPR-Cas susceptible virus, forces rapid spacer acquisition in the *Sulfolobus islandicus* LAL14/1 CRISPR arrays. Spacer acquisition was observed as early as 30 minutes post infection, with the newly acquired spacers uniformly distributed across the genome of the virus. Although the newly acquired spacers were predominantly effective only against the CRISPR-Cas susceptible mutant virus, we were able to isolate a host mutant with a novel spacer which provides immunity against the multiple Acr encoding wildtype virus, *Sulfolobus islandicus* rod-shaped virus 2 (SIRV2).

**Figure 1. Rapid spacer acquisition in S. islandicus LAL14/1 from SIRV2M.**
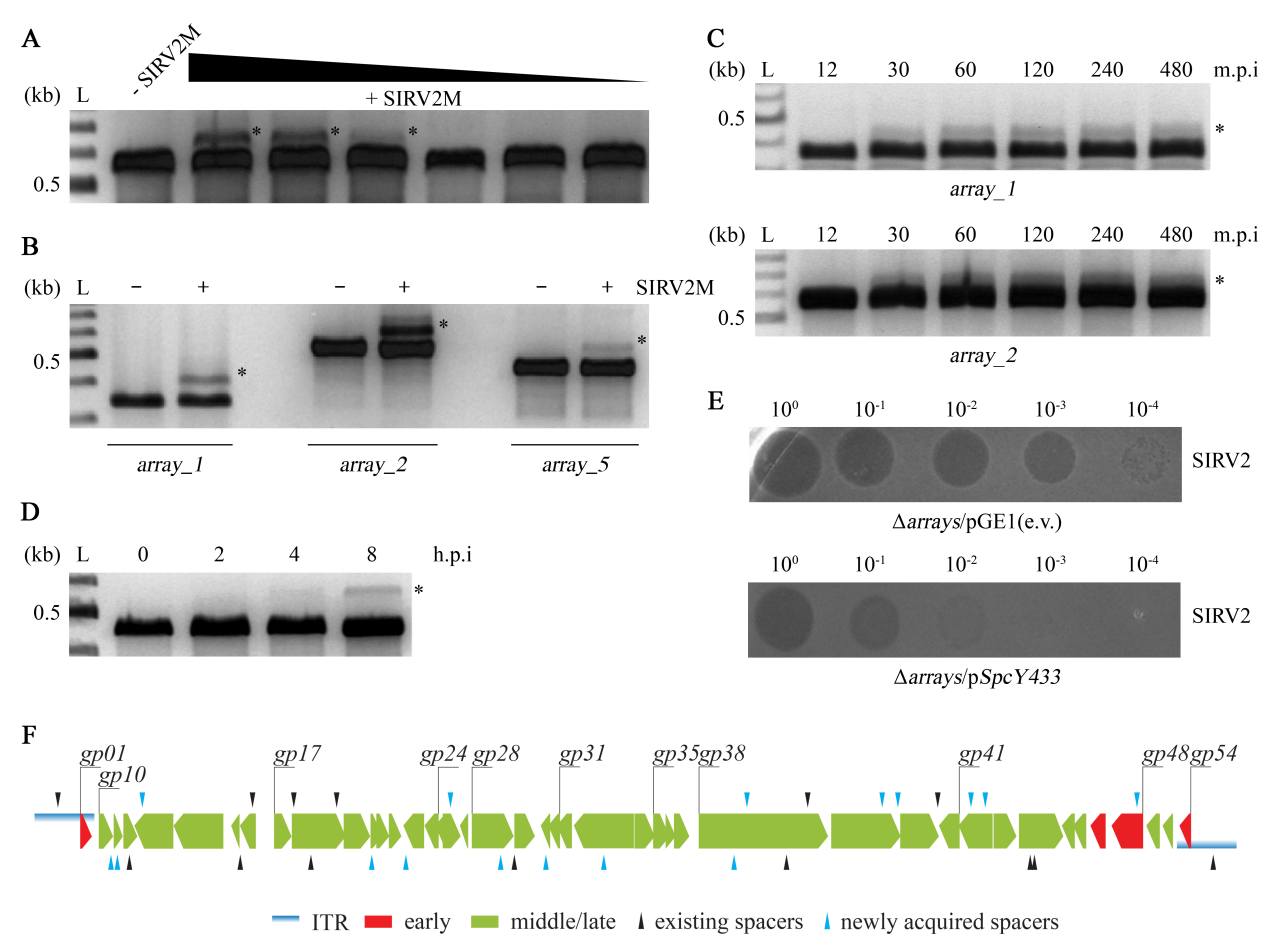
**A.** Acquisition within *S. islandicus* LAL14/1 array_2 after SIRV2M infection at different MOIs. Total DNA from cell cultures either uninfected (- SIRV2M) or SIRV2M infected (+ SIRV2M) at MOIs 50, 25, 5, 5 X 10 ^-1^ , 5 X 10 ^-2^ and 5 X 10 ^-3^ was extracted 4 hour post infection (h.p.i) and used as template to amplify the leader repeat junction of *array_2* using the primer pair Array2_LR_For/Array2_LR_Rev. **B** . Spacer acquisition in *S. islandicus* LAL14/1 *array_1* , *array_2* and *array_5* . Total DNA from cultures without (-) or with (+) SIRV2M infection (MOI of around 20) was extracted 2 h.p.i and subject to PCR amplification using primer pairs specific for the leader-repeat junctions of the arrays, Array1_LR_For/Array1_LR_Rev for *array_1* , Array2_LR_For/Array2__LR_Rev for *array_2* and Array5_LR_For/Array5_LR_Rev for *array_5* . **C** . Temporal analysis of spacer acquisition in *array_1* and *array_2* . SIRV2M infected cultures (MOI = 20) were sampled at the indicated time points and analyzed by PCR. m.p.i = minutes post infection **D** . Spacer acquisition into artificial leader repeat junction. *S. islandicus* LAL14/1 with plasmid borne leader repeat sequence was infected with SIRV2M (MOI = 20) and sampled at various time points. The leader repeat junction on the plasmid was PCR analyzed. **E** . Spot assay comparing the infectivity of SIRV2 in *S. islandicus* LAL14/1 Δ *arrays* pGE1 (empty vector) and *S. islandicus* LAL14/1 Δ *arrays* p *SpcY433* . **F** . Illustration of protospacers in SIRV2M. Distribution of protospacers corresponding to preexisting spacers in *S. islandicus* LAL14/1 (black arrows) or new spacers (blue arrows) sequenced here (Extended Data) are shown on a genomic map of SIRV2M. Early - SIRV2 early expressed genes, middle/late - SIRV2 middle/late expressed genes. Horizontal bars in blue - inverted terminal repeat (ITR). **A-D** . L - DNA ladder. PCR bands containing new spacers are indicated by *.

## Description

CRISPR-Cas adaptation or spacer acquisition involves the integration of a DNA fragment from an invading MGE into the leader-repeat junction of the CRISPR array. Transcription of the array and subsequent processing provides the template, crRNA, necessary for the identification and targeting of the invader. In archaea, spacer acquisition from conjugative plasmids and viruses was observed 3-12 days post coinfection with *Sulfolobus* monocaudavirus (SMV1) in *Sulfolobus* (Erdmann & Garrett, 2012; Erdmann et al., 2013). Spacer acquisition was also observed upon overexpression of the transcriptional regulator, Csa3a, predominantly from the *Sulfolobus* genome (Liu et al., 2015). Here we present a system with accelerated spacer uptake that could be an ideal choice for the study of acquisition in *Sulfolobus* .

Previously, we isolated a mutant SIRV2 virus with a 3.9 kbp deletion on the left terminus of the wildtype genome upon propagation in a CRISPR deficient host (He et al., 2018). The mutant virus, SIRV2M, lacking the subtype I-D inhibitor AcrID1, is sensitive to targeting by the CRISPR-Cas systems of the wildtype host *S. islandicus* LAL14/1. This feature allowed us to study possible spacer uptake from SIRV2M in the wildtype host. Upon infection with a range of MOIs, we observed spacer uptake 2 hours post infection within the CRISPR *array_2* at MOIs greater than 5 (Figure 1A). *S. islandicus* LAL14/1 encodes 5 CRISPR arrays classified into subtype I-A leader-repeat ( *array_1* and *array_2* ) and subtype I-D leader-repeat ( *array_3* , *array_4* and *array_5* ) (Jaubert et al., 2013). Spacer uptake was observed in at least one of the subtype I-A and one of the subtype I-D arrays, indicating that both acquisition modules in *S. islandicus* LAL14/1 were activated upon high MOI infection (Figure 1B). Furthermore, sampling at earlier time points showed that spacer uptake was initiated as early as 30 minutes post infection and acquisition intensity increased at later time points (Figure 1C). SIRV2 specific spacer uptake was also seen on a plasmid engineered to encode the subtype I-A ( *array_2* ) leader-repeat sequence (Figure 1D).

Next, we tried to isolate *S. islandicus* LAL14/1 strains with new spacers in the CRISPR arrays. To achieve this, cells infected with SIRV2M at high-MOI for ~20 hours were washed to remove any extracellular virus, plated and screened for spacer acquisition in single colonies. Four positive colonies with single spacer acquisition and one colony with double spacer acquisition were isolated. As with the WT host, all the five colonies were immune to infection by the mutant virus whereas one of the five isolates, *S. islandicus* LAL14/1 CR1Y433 gained immunity to the wildtype virus, SIRV2. Interestingly, the new spacer, Y433 (Extended Data), of the isolate CR1Y433 matched in sequence the transcript of the SIRV2 early gene *gp48* i.e., *acrIIIB1* . In order to verify the role of Y433 in CR1Y433 immunity against SIRV2, we constructed a plasmid based mini-CRISPR array carrying Y433 under the control of an arabinose promoter (p *SpcY433* ). *S. islandicus* LAL14/1 Δ *arrays* p *SpcY433* showed complete resistance to the wildtype virus SIRV2 confirming that the resistance gained in the isolate CR1Y433 was solely due to the presence of the new spacer Y433 (Figure 1E). In total, we sequenced 16 novel spacers acquired on the CRISPR arrays either on the host genome or on the plasmid encoding *array_2* leader-repeat (Extended Data). The newly acquired spacers were evenly distributed across the genome of the SIRV2M virus with no strand bias, implying a naive spacer acquisition (Figure 1F). On average, the spacers were 41 bps in length, with the protospacer adjacent motif (PAM) predominantly CCN for subtype I-A spacers (Extended Data).

Previously, naive spacer acquisition was proposed to occur from inactivated viruses as demonstrated with a replication deficient bacteriophage (Hynes et al., 2014). In accordance with this work, we propose here that naturally occurring Acr deficient viruses, generated upon infection of CRISPR-deficient hosts, could be targets for naive spacer uptake in CRISPR-active hosts. As an overexpression of the HEPN domain containing transcription regulator Csa3a was found to induce spacer acquisition in *Sulfolobus* CRISPR I-A loci (Liu et al., 2015), the accelerated spacer uptake observed here could be due to potential activation of the corresponding HEPN regulatory proteins of the I-A and I-D systems (Csa3a and Csa3, respectively) upon continuous SIRV2M infection at high MOIs.Despite encoding 13 spacers matching SIRV2 genome (Jaubert et al., 2013), the wild-type host is compelled to reinforce its CRISPR-Cas immune system with additional spacers to overcome continuous virus infection. Previously, we have also demonstrated that early gene targeting by subtype III-B systems is immune to inhibitory activity of AcrIIIB1 (Bhoobalan-Chitty et al., 2019). Here, isolation of *S. islandicus* LAL14/1 CR1Y433 demonstrates that acquisition of a spacer targeting early viral gene(s) can indiscriminately protect the host from viruses carrying multiple Acrs, including an inhibitor of type III system. Similar occurrences in a natural environment would lead to complete eradication of viruses. Therefore, a lack of spacers targeting early SIRV2 genes in *S. islandicus* LAL14/1 could be either due to rapid mutations within the protospacer regions or the presence of an inhibitor of spacer acquisition among SIRV2 early genes, which are absent in the mutant virus, SIRV2M.

## Methods

All *S. islandicus* LAL14/1 and Δ *arrays* liquid cultures were grown at 78℃, 200 rotations per minute. The *E. coli* / *Sulfolobus* shuttle vector pEXA was used for cloning of the *array_2* leader repeat sequence into *S. islandicus* LAL14/1. Electroporation of plasmid into *Sulfolobus* and virus titre estimations were performed as described earlier (Alfastsen et al., 2021). The mini-CRISPR loci plasmid, p *SpcY433* transcribing the spacer Y433 targeting *SIRV2gp48* was constructed as described earlier (Peng et al., 2015) using primers Y433_Spc_For and Y433_Spc_Rev.

**Spacer acquisition assay**

Overnight *Sulfolobus* cultures were transferred to fresh medium at OD _600_ = 0.05 and allowed to grow until the OD _600_ reached between 0.1 and 0.2. Specified amount of virus supernatant was transferred into the cultures to achieve the desired MOI. At the specified time intervals 10 ml of cell culture was withdrawn and centrifuged at 6300 x g for 6 minutes. The pellet was washed twice with medium salts and resuspended in TL buffer. Total genomic DNA was extracted using the E.Z.N.A Tissue DNA kit (Omega BIO-TEK) following the manufacturer instructions. The extracted genomic DNAs were utilized as templates in the proceeding PCR reactions. For sequencing of novel spacers, *array_2* was amplified with primers, Array2_LR_SphI_For and Array2_LR_NotI_Rev, the expanded bands of size larger than the wildtype arrays were gel extracted, restriction digested and cloned into the pEXA plasmid. Plasmids carrying new spacers were sequenced using plasmid specific sequencing primers.

**Single colony isolation**

High MOI infection of *S. islandicus* LAL14/1 was performed as defined in the spacer acquisition assay. The withdrawn samples were washed thrice with either medium salts or *Sulfolobus* medium (Zillig et al., 1993; Alfastsen et al., 2021) to remove any virus present in the supernatant. Serial dilutions of the infected cells were plated onto a 2X SCV/gelrite plate and incubated for 7-10 days at 78℃. The single colonies were resuspended in medium salts, spotted onto new 2X SCV/gelrite plates and incubated for 3 days at 78℃. The spots were then transferred into liquid SCV medium. Genomic DNA, extracted from the single colonies, was used as templates in PCR to detect clones with new spacers acquired into the CRISPR arrays.

## Reagents

Table 1: primers used in this study

**Table:** 

	Oligonucleotide	Sequence (5′ - 3′)
pEXA- *array2L/R*	Array2_LR_SphI_For	TA ***GCATGC*** TCCCGTATACGATCCTTGT
	Array2_LR_NotI_Rev	ATT ***GCGGCCGC*** TAGTGCTTCCTTTGT CATTC
Primer pairs for amplification of leader-repeat sequence	Array1_LR_For	TTAGCGAAGAAGTGAAAGATCA
	Array1_LR_Rev	TTTTGATTACTTTCGAGGAACTC
	Array2_LR_For	TGAAGCCTCCTAACCTGTCTA
	Array2_LR_Rev	GCTATGCAAAATGTAAGTCAAAA
	Array5_LR_For	AAGCGCGTTGAACTAGAATAA
	Array5_LR_Rev	TGTTGTTACGAGTTTGCATTT
p *SpcY433*	Y433_Spc_For	AAAGACCTGCATTACCTGTACACATC CTTCCTGTGTCATCCCTGACC
	Y433_Spc_Rev	TAGCGGTCAGGGATGACACAGGAAG GATGTGTACAGGTAATGCAGGT

Italicised nucleotides correspond to restriction sites utilised for cloning and sequencing of *array_2* .

## Extended Data

Description: List of spacer sequences newly acquired upon infection with SIRV2M (column 1) and their PAM sequence (column 2). The CRISPR-Cas subtypes capable of utilizing each new spacer is specified (column 3) along with the location of the spacer in the SIRV2 genome (column 4). Spacer complementarity to sense strand (column 1) is especially important for the transcription dependent targeting of subtype III-B CRISPR-Cas systems.. Resource Type: Text. DOI: 10.22002/che7n-2dw90
